# The contribution of at-home and away-from-home food to dietary intake among 2–13-year-old Mexican children

**DOI:** 10.1017/S1368980016002196

**Published:** 2016-09-09

**Authors:** Lindsey Smith Taillie, Myriam C Afeiche, Alison L Eldridge, Barry M Popkin

**Affiliations:** 1 Department of Nutrition, Gillings School of Global Public Health and School of Medicine, University of North Carolina, Chapel Hill, CB # 8120 University Square, Chapel Hill, NC 27516–3997, USA; 2 Nestlé Research Center, Lausanne, Switzerland

**Keywords:** Eating location, Energy intake, Child diet, Fast food, Latin America

## Abstract

**Objective:**

Away-from-home foods have been shown to have lower nutritional quality and larger portion sizes than many foods prepared at home. We aimed to describe energy and nutrient intakes among 2–13-year-old Mexican children by eating location (at home and away from home), overall, by socio-economic status (SES) and by urbanicity.

**Design:**

Dietary intake was collected via one 24 h recall in the 2012 Mexican National Health and Nutrition Survey (ENSANUT). Location was reported for each food consumed. Results were adjusted for sex, day of recall, region, weight status, SES and urbanicity.

**Setting:**

Mexico (nationally representative).

**Subjects:**

Children aged 2–5 years (*n* 1905) and 6–13 years (*n* 2868).

**Results:**

Children consumed the majority of daily energy at home (89% of 2–5-year-olds; 82 % of 6–13-year-olds). The most common away-from-home eating location was school (22 % of 2–5-year-olds; 43 % of 6–13-year-olds), followed by the street (14 % of 2–5-year-olds; 13 % of 6–13-year-olds). The most common foods consumed away from home were wheat/rice and corn mixed dishes, sugar-sweetened beverages, pastries/candy/desserts, milk (2–5-year-olds only) and salty snacks (6–13-year-olds). Multivariate models showed that high-SES 2–5-year-olds consumed 14 % of daily energy away from home *v*. 8 % among low-SES 2–5-year-olds, and high-SES 6–13-year-olds consumed 21 % of daily energy away from home *v*. 14 % among low-SES 6–13 year-olds. There were no differences by urban residence.

**Conclusions:**

Among Mexican children, most foods and beverages were consumed at home. However, the percentage of foods consumed or purchased away from home increased with age and with SES.

Overweight and obesity are a major health concern in Mexico, with an estimated prevalence of 9 % among children of pre-school age (0–59 months)^(^ ^1^ ^)^ and 35 % among school-age children (5–11 years)^(^ ^2^ ^)^. Identifying dietary behaviours or environments that are linked to excess energy intake is a critical step in understanding where along the pathway policies or interventions can help prevent the continued rise of obesity. The away-from-home food environment is one potential area for improvement, as previous research has found that away-from-home food tends to have lower nutritional quality, including more energy, total fat and saturated fat, as well as less dietary fibre, vitamin C, Ca and Fe^(^ ^3^ ^,^ ^4^ ^)^. Larger portion sizes of away-from-home foods also contribute to increased energy intake^(^ ^5^ ^–^ ^8^ ^)^.

In the USA, away-from-home consumption accounts for 31 % of total energy among 2–5-year-olds and 36 % among 6–11-year-olds^(^ ^9^ ^)^. Recent research has also found that energy from stores, fast foods and school, all are comprised of about 32–35 % ‘empty’ calories from solid fats and added sugars (SoFAS)^(^ ^10^ ^)^.

Little is known about away-from-home food and beverage intakes in Mexico and how this contributes to food group and total daily intakes. Quantifying the extent to which children consume daily energy away from home and the dietary quality associated with away-from-home foods and beverages, including in schools, can help pinpoint which aspects of the food environment could be improved. This is especially true for households with lower socio-economic status (SES), which tend to have poorer diet quality and poorer health outcomes, and urban households, which in many other countries tend to have a higher intake of away-from-home foods^(^ ^4^ ^)^. The objective of the present study was first to describe the energy and nutrient intakes of children aged 2–5 years and 6–13 years by eating location (at home and away from home), including the nutritional quality of foods consumed and the types of foods consumed. In addition, mean energy intake from at-home and away-from-home locations by SES and urbanicity was evaluated.

## Methods

### Study population

The 2012 Encuesta Nacional de Salud y Nutrición (ENSANUT; Mexican National Health and Nutrition Survey) is a cross-sectional, multistage, stratified and cluster-sampled survey of 50 528 households (response rate 87 %) that was conducted by Mexico’s Instituto Nacional de Salud Pública (National Institute of Public Health) between October 2011 and May 2012. The primary sampling units were geostatistical basic areas, or Mexico’s census units. The survey was designed to be representative at the regional and state level, including rural and urban areas within each state, and with oversampling of low-SES sub-populations. The sample was designed to achieve adequate samples by age group, including pre-school children (age 0–4 years), elementary-school children (5–9 years) and adolescents (10–19 years)^(^ ^11^ ^,^ ^12^ ^)^. In the current paper, the age categories were re-grouped to examine 2–5-year-olds and 6–13-year-olds. Trained interviewers were used to perform all assessments, including dietary interviews and anthropometry, in respondents’ homes.

### Dietary assessment

To capture information on the types and amount of foods and beverages consumed during the preceding 24 h, dietary intake data were collected on a random sub-sample across all ages (*n* 9937) using one 24 h recall administered via the Automated 5-step Multiple Pass Method^(^ ^13^ ^)^. This 24 h recall method was adapted to the Mexican context^(^ ^14^ ^)^ by the Mexico National Institute of Public Health, including translation to the Spanish language and adaptations to reflect unique characteristics of food intake in Mexico (e.g. characteristics of purchased foods (raw or processed, packaged or unpackaged, frozen or not frozen), location of intake and portion sizes).

For children younger than 15 years old, the primary household meal preparer reported food intake, with children confirming foods and beverages consumed while not in the presence of the primary meal preparer (e.g. at school). Interviewers used tools to aid in portion size estimation, including photographs of commonly consumed foods, a food scale, a measuring cup and a serving spoon. When the weight or volume was not reported, grams or millilitres of the item consumed were imputed by age group, region of residence and mealtime. Participants reported the eating occasion for each item consumed, including breakfast (first meal of the day), lunch (often the main meal, consumed between noon and mid-afternoon), dinner (evening meal), *almuerzo* (which is a meal that occurs after breakfast, typically late morning or noon) and as a snacking occasion (any food or beverage contributing >0 kJ that was consumed between the customary mealtimes).

Whole foods were reported as consumed (i.e. banana; yoghurt). Mixed dishes were reported as a single item and then disaggregated into component ingredients using either a standard recipe (when the mixed dish was consumed away from home or the specific proportion of ingredients was partially or wholly unknown) or a custom recipe (when the mixed dish was prepared at home and ingredients were known). A standard recipe is based on a weighted average of typical recipes (comprised of ingredients), whereas a custom recipe reflects ingredient by ingredient what a particular mixed dish contained. The food groups used in the study were based on the food groups used in the 2008 US Feeding Infants and Toddlers Study^(^ ^15^ ^)^. Two trained Mexican dietary research specialists and a Nestlé nutrition scientist modified existing groups and created additional groups to reflect foods consumed by children in Mexico, such as the addition of tortillas.

The most recent Mexican food composition tables were used^(^ ^14^ ^)^. To calculate SoFAS, each food was linked at the ingredient level (single foods, standardized recipes) or dish level (custom recipes) to the US Department of Agriculture’s National Nutrient Database for Standard Reference^(^ ^16^ ^)^, and then further linked to the MyPyramid Equivalents Database^(^ ^17^ ^)^.

### Categorization of at home and away from home

Respondents reported an eating location (i.e. where the food was consumed) for all foods and beverages consumed. Items were classified as ‘at home’ if they were consumed in the home; otherwise, the location was classified as ‘away from home’ and included work, school, in transportation, restaurant, sports arena, street vendor, *puesto ambulante* (similar to street vendor) and other. The source of food (i.e. where the food was obtained) was reported directly only for foods for which a standardized recipe was reported, with just limited options available (home, supermarket, restaurant or fast food). Thus we examined only the location of food consumption and not the location of food purchased.

### Additional covariates

Urbanicity was based on the population and categorized as rural or urban (>2500 residents). SES was based on household assets (pre-calculated in ENSANUT 2012) and grouped into tertiles (low, medium and high) for the population. Overweight and obesity was classified using the International Obesity Task Force cut-off points^(^ ^18^ ^,^ ^19^ ^)^. Of 5027 children aged 2–13 years, 254 were excluded because they had missing information on weight status (5 % of the sample). Age was stratified by pre-primary school age (2–5 years, *n* 1905) and primary school age (6–13 years, *n* 2868; total analytical sample, *n* 4773).

### Statistical analysis

First, sociodemographic characteristics were summarized by eating location using *t* tests for means. Then, the mean daily per capita intakes of energy, macronutrients and micronutrients as well as the mean daily per capita energy intakes from food groups were examined by eating location. To examine whether eating location differed by urbanicity and SES, we used multivariate linear regression stratified by age group (2–5 years *v*. 6–13 years) controlling for key covariates, including day of recall (weekday *v*. weekend), sex, region (North, Central, Mexico City *v*. South) weight status (normal weight *v*. overweight/obese), urbanicity (in SES models) and SES (in urbanicity models). For the multivariate analysis, margins commands were used to predict mean energy intake for each level of consumption status of each key eating occasion, adjusted for the aforementioned variables. Finally, we conducted a sensitivity analysis comparing the sociodemographic characteristics and mean daily energy intakes for children excluded due to missing weight status and those included. Statistical analyses were conducted using the statistical software package Stata version 14. All analyses were adjusted to be nationally representative using Stata’s svy commands and stratum-specific probability weights supplied by ENSANUT to account for the complex survey design. Statistical significance for multivariate analyses was defined at *P*<0·05.

## Results

Mean daily energy intake was 6251 (se 96) kJ/d (1494 (se 23) kcal/d) for 2–5-year-old children and 7979 (se 100) kJ/d (1907 (se 24) kcal/d) for 6–13-year-old children (Table 1). Overall, the majority of daily energy consumed was at home, although this decreased with age (89 % for 2–5-year-olds *v*. 82 % for 6–13-year-olds, *P*<0·05). Boys consumed significantly more energy than girls, but away-from-home consumption was similar between boys and girls. Children living in the North consumed more energy daily than those in the South, and those living in the Central region and Mexico City consumed more energy away from home. There were no differences in total daily energy intake by urbanicity. There was a positive association between SES and total daily energy intake. Away-from-home food intake was higher on weekdays (20 % of daily energy) *v*. weekends (9 % of daily energy, *P*<0·05).

**Table 1:** Mean per capita total daily energy intake, percentage of consumers and mean per capita daily energy intake from at-home and away-from-home eating locations among nationally representative Mexican children aged 2–13 years, 2012 Encuesta Nacional de Salud y Nutrición (ENSANUT; Mexican National Health and Nutrition Survey)

		Mean per capita daily energy (kcal/d)†							
		Total		At home			Away from home		
	*n* ‡	Mean	se	% of consumers	Mean	se	% of consumers	Mean	se
Age									
2–5 years (ref.)	1905	1494	23	99	1333	24	37	161	10
6–13 years	2868	**1907**	24	99	**1549**	23	**56**	**359**	15
Sex									
Male (ref.)	2415	1854	28	99	1565	28	48	289	18
Female	2358	**1712**	23	99	**1403**	23	52	310	14
Region									
South (ref.)	1735	1756	28	100	1506	26	41	251	16
Central	1734	1726	28	99	**1399**	28	**53**	**327**	18
North	1077	**1867**	40	**99**	1571	39	**52**	295	19
Mexico City	227	1853	74	99	1499	69	**60**	**355**	49
Urbanicity									
Rural (ref.)	1869	1759	29	100	1489	29	42	270	17
Urban	2904	1793	24	99	1481	23	**53**	312	15
Weight status									
Normal weight (ref.)	3642	1760	21	99	1488	20	47	272	11
Overweight/obese	1131	**1852**	36	100	1471	35	**58**	**381**	30
Socio-economic status									
Lowest tertile (ref.)	1800	1717	30	100	1505	30	37	212	15
Middle tertile	1707	**1807**	32	**99**	1486	34	**53**	**320**	16
Highest tertile	1266	**1826**	36	99	1457	32	**60**	**369**	27
Dietary recall on weekday									
Yes (ref.)	3512	1788	22	99	**1439**	20	57	349	13
No	1261	1769	37	99	1611	39	**28**	**158**	17
Eating occasion, per capita									
Breakfast (ref.)	3953	380	7	74	328	7	10	52	5
*Almuerzo*	2254	220	8	26	125	7	19	95	6
Lunch	4322	521	12	83	473	12	8	48	4
Dinner	3888	360	8	79	345	8	3	15	2
Total snacks	3358	302	11	57	212	8	25	89	8

ref., reference category. Bold indicates that the percentage consumers of that eating location or food source or the mean per capita daily energy from that eating location or source was significantly different from that of the referent group, via the *χ* ^2^ test for percentage of consumers or the *t* test for means, respectively (*P*<0·05).

† To convert kilocalories to kilojoules, multiply kcal value by 4·184.

‡ Sample sizes for demographics are based on all respondents with non-missing values on the model variables used in the table. Sample sizes for eating occasion are those who consumed >0 kcal during the eating occasion.

As a result of the much higher energy intake from the at-home eating location, at-home foods were the largest contributor not only to daily energy but also to all macro- and micronutrient intakes (Table 2). This included SoFAS, with 88 % of total daily SoFAS intake consumed at home for 2–5-year-olds and 80 % of total daily SoFAS intake consumed at home for 6–13-year-olds.

**Table 2:** Mean per capita daily micro- and macronutrient intakes and percentage of mean per capita daily micro- and macronutrient intakes from at-home and away-from-home eating locations, according to age, among nationally representative Mexican children aged 2–13 years, 2012 Encuesta Nacional de Salud y Nutrición (ENSANUT; Mexican National Health and Nutrition Survey)

	2–5 years						6–13 years					
	Total intake		At home		Away from home		Total intake		At home		Away from home	
	Mean	se	%	se	%	se	Mean	se	%	se	%	se
Macronutrients												
Energy (kcal/d)†	1494	23	89	0·7	11	0·7	1908	24	82	0·7	18	0·7
Total fat (kcal/d)	502	10	89	0·7	11	0·7	645	11	81	0·7	19	0·7
Saturated fat (kcal/d)	196	4	89	0·8	11	0·8	233	4	82	0·7	18	0·7
MUFA (kcal/d)	170	4	89	0·8	11	0·8	220	4	81	0·8	19	0·8
PUFA (kcal/d)	110	3	88	0·9	12	0·9	155	3	80	0·8	20	0·8
Solid fat (kcal/d)	225	8	89	0·8	11	0·8	262	9	82	0·8	18	0·8
Carbohydrate (kcal/d)	812	15	89	0·7	11	0·7	1048	13	82	0·6	18	0·6
Total sugar (kcal/d)	341	8	88	0·7	12	0·7	371	7	80	0·7	20	0·7
Added sugars (kcal/d)	188	6	86	1·0	14	1·0	221	5	78	0·9	22	0·9
SoFAS (kcal/d)	413	11	88	0·7	12	0·7	483	12	80	0·8	20	0·8
Protein (kcal/d)	207	4	90	0·7	10	0·7	252	4	84	0·6	16	0·6
Dietary fibre (g/d)	15	1	88	0·9	12	0·9	21	0	82	0·7	18	0·7
Antioxidants												
Vitamin C (mg/d)	100	4	87	0·9	13	0·9	120	5	79	0·8	21	0·8
Vitamin E (mg/d)	6	0	89	0·8	11	0·8	8	0	80	0·8	20	0·8
B vitamins												
Thiamin (mg/d)	1	0	90	0·8	10	0·8	4	2	84	0·6	16	0·6
Riboflavin (mg/d)	1	0	90	0·7	10	0·7	2	0	85	0·6	15	0·6
Niacin (mg/d)	13	0	90	0·8	10	0·8	16	0	85	0·6	15	0·6
Vitamin B_6_ (mg/d)	1	0	90	0·8	10	0·8	3	1	84	0·6	16	0·6
Folate (μg DFE/d)	175	8	89	0·9	11	0·9	206	6	83	0·7	17	0·7
Vitamin B_12_ (μg/d)	3	0	90	0·8	10	0·8	4	0	85	0·7	15	0·7
Bone-related nutrients												
Ca (mg/d)	843	20	90	0·7	10	0·7	862	17	83	0·7	17	0·7
P (mg/d)	1017	20	90	0·8	10	0·8	1323	19	85	0·6	15	0·6
Mg (mg/d)	243	5	89	0·8	11	0·8	327	5	83	0·6	17	0·6
Vitamin D (μg/d)	86	5	90	1·0	10	1·0	89	6	83	1·0	17	1·0
Other micronutrients												
Vitamin A (μg RE/d)	230	8	90	0·9	10	0·9	238	9	84	0·9	16	0·9
Vitamin K (μg/d)	43	2	88	0·9	12	0·9	60	3	80	0·9	20	0·9
Fe (mg/d)	11	0	90	0·7	10	0·7	13	0	84	0·6	16	0·6
Zn (mg/d)	9	0	90	0·7	10	0·7	10	0	84	0·6	16	0·6
Na (mg/d)	2040	55	89	0·8	11	0·8	2735	67	82	0·7	18	0·7
K (mg/d)	1947	48	89	0·8	11	0·8	2327	39	83	0·6	17	0·6

SoFAS, solid fats and added sugars; DFE, dietary folate equivalents; RE, retinol equivalents.

†To convert kilocalories to kilojoules, multiply kcal value by 4·184.

**Fig. 1:**
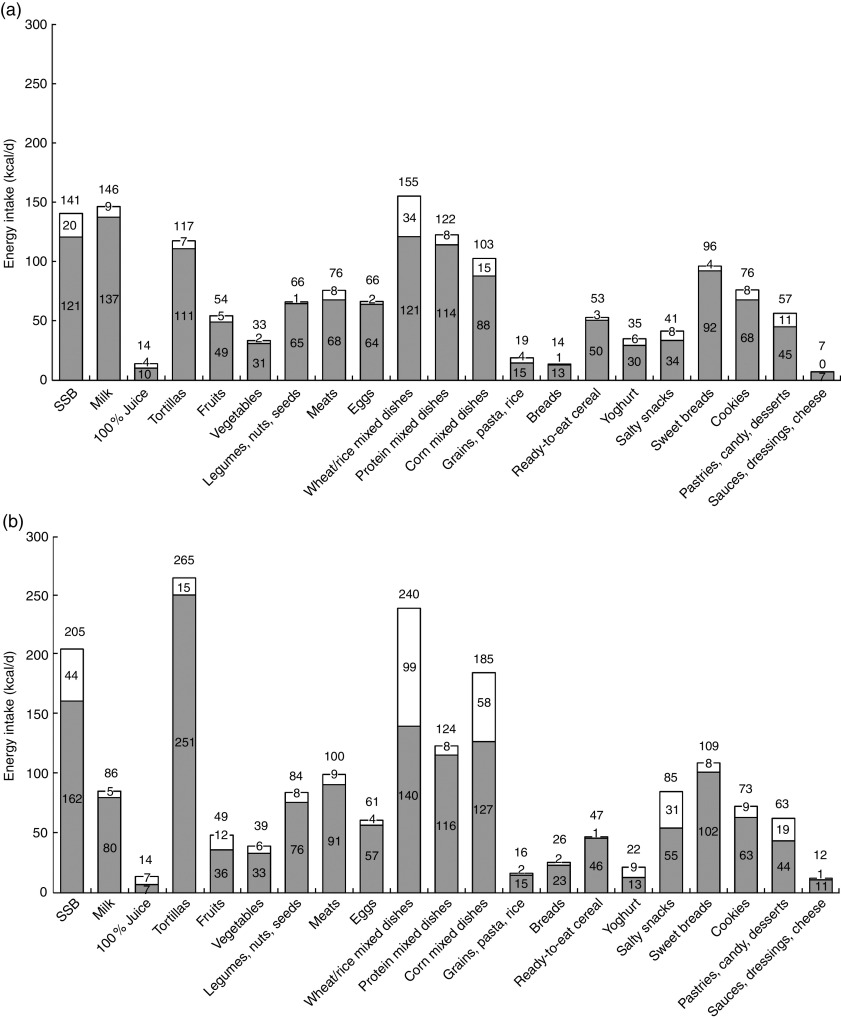
Mean per capita daily energy intake† from food groups by eating location (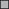, at home; 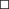, away from home) among nationally representative Mexican children aged (a) 2–5 years (*n* 1905) and (b) 6–13 years (*n* 2868), 2012 Encuesta Nacional de Salud y Nutrición (ENSANUT; Mexican National Health and Nutrition Survey). †To convert kilocalories to kilojoules, multiply kcal value by 4·184 (SSB, sugar-sweetened beverages)

With regard to energy contribution, wheat/rice mixed dishes, corn mixed dishes, sugar-sweetened beverages (SSB) and pastries/candy/desserts were the top contributors to away-from-home food intake for both age groups, while milk was a top five contributor for 2–5-year-olds and salty snacks for 6–13-year-olds (Fig. 1(a) and 1(b)).

**Fig. 2:**
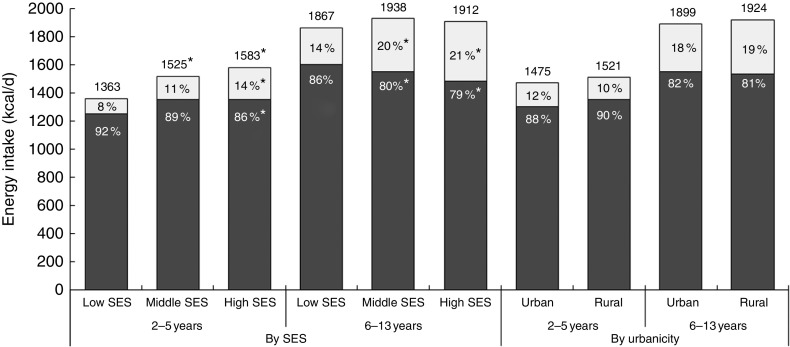
Adjusted predicted mean per capita daily energy intake† by eating location (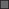, at home; 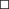, away from home), socio-economic status (SES) and urbanicity among nationally representative Mexican children aged 2–5 years (*n* 1905) and 6–13 years (*n* 2868), 2012 Encuesta Nacional de Salud y Nutrición (ENSANUT; Mexican National Health and Nutrition Survey). Models adjusted for sex, day of recall, overweight status, region, and urbanicity (in SES models) or SES (in urbanicity models). *Percentage energy intake from eating location was significantly different from that of low SES: *P*<0·05. †To convert kilocalories to kilojoules, multiply kcal value by 4·184

The models of adjusted predicted mean energy intake showed a positive association between SES and total energy intake for younger but not older children (5703 (se 155) kJ/d (1363 (se 37) kcal/d) for low-SES 2–5-year-olds *v*. 6623 (se 188) kJ/d (1583 (se 45) kcal/d) for high-SES 2–5-year-olds, *P*<0·05 and 7812 (se 184) kJ/d (1867 (se 44) kcal/d) for low-SES 6–13-year-olds *v*. 8000 (se 188) kJ/d (1912 (se 45) kcal/d) for high-SES 6–13-year-olds, NS; Fig. 2). Low-SES children also ate a greater percentage of daily energy at home (92 and 86 % for 2–5-year-olds and 6–13-year-olds, respectively) compared with high-SES children (86 and 79 %, respectively, *P*<0·05). Despite differences in unadjusted analyses, there were no major differences in total energy intake or eating location by urbanicity.

The most common away-from-home eating location was school (22 % of 2–5-year-olds; 43 % of 6–13-year-olds), followed by street vendors (14 % of 2–5-year-olds; 13 % of 6–13 year-olds; see online supplementary material, Supplemental Table 1). The school eating location accounted for 6 and 13 % of mean daily energy among 2–5-year-old and 6–13-year-old children, respectively, and was driven predominantly by intake of *almuerzo*. Snacking occasions were the biggest contributor to street vendor intake.

The sensitivity analysis comparing children excluded for missing weight status *v*. included children with complete information on weight status (see online supplementary material, Supplemental Table 2) found that 2–5-year-old children with missing weight status consumed less energy in total (5627 (se 238) kJ/d (1345 (se 57) kcal/d)) and at home (4841 (se 264) kJ/d (1157 (se 63) kcal/d)) compared with 2–5-year-olds with non-missing weight status (total energy: 6251 (se 96) kJ/d (1494 (se 23) kcal/d); at-home energy: 5577 (se 100) kJ/d (1333 (se 24) kcal/d, *P*<0·05 for each comparison), but there were no differences in away-from-home energy. For 6–13-year-olds, there were no differences for total daily, at-home or away-from-home energy intake. Children with missing weight status were more likely to be in the highest tertiles of SES (37 *v*. 27 %, *P*<0·05), but there were no differences by urbanicity.

## Discussion

Among Mexican children aged 2–13 years, the majority of food intake was consumed at home. Younger and older children consumed only 11 and 19 % of energy away from home, respectively, which is substantially lower than in the USA, where children aged 2–12 years consume between 29 and 35 % of energy away from home^(^ ^20^ ^,^ ^21^ ^)^. In general, little is known about away-from-home food intake in Latin America, making comparisons difficult, but evidence from Brazil shows that among individuals aged 10 years or older, 43 % consumed at least one food item away from home daily (18 % of total daily intake)^(^ ^22^ ^)^.

In line with energy contributions, foods consumed at home contributed most of children’s daily macro- and micronutrients, including energy from SoFAS. Wheat and rice mixed dishes, and corn mixed dishes, including sandwiches, tacos, enchiladas and pasta dishes, were among the biggest contributors to away-from-home foods. SSB and pastries, candy and other desserts were also top contributors. Children aged 6–13 years consumed a disproportionate amount of energy from these food groups away from home: for example, they consumed 36 % of salty snacks and 30 % of pastries, candies and desserts away from home (compared with 18 % of overall energy consumed away from home). On the other hand, children aged 6–13 years also consumed disproportionately more yoghurt (40 %), fruit (25 %), 100 % fruit juice (48 %) and vegetables (20 %) away from home. While research from Brazil shows that that away-from-home foods tend to have high energy density (e.g. baked and fried snacks, pizza, soft drinks, sandwiches and sweets)^(^ ^22^ ^)^, the present results show that for Mexican children, away-from-home foods can be a source of both staple foods as well as snack-type foods and SSB.

This combination of both healthy and less-healthy away-from-home foods may be because the majority of away-from-home foods were consumed at school. While away-from-home food intake increased with age, this was driven predominantly by higher food intake at school during *almuerzo*, the late-morning meal (in Mexico, it is common for children to consume lunch, often the biggest meal of the day, upon returning home from school). However, it is important to consider that food consumed at school may be obtained from various sources (e.g. it could be prepared at home, in the school, or at a restaurant or other vendor near the school) and these different sources could be linked to nutritional quality. One nationally representative study conducted in 2013 found that 41 % of 9–10-year-old children brought food from home to school, while 29 % purchased food at school, and 30 % did both (N Lopez-Olmedo, unpublished results). An additional study of eight public schools in Tijuana found that while virtually all pre-school children consumed foods prepared at home at school, this declined as children aged, whereas the purchase of unhealthy foods both inside and outside the school increased with age^(^ ^23^ ^)^. The same study found that whereas foods from home tended to be healthier, with fruits and vegetables among the most commonly brought-from-home items, foods purchased both in and outside the school comprised largely ‘fried foods, soft drinks, ice cream, yogurt high in sugar, pastries, cookies, processed soups and sweets’, as well as burgers, pizza, burritos and quesadillas.

It was not possible to ascertain whether the foods brought from home were more or less healthy than those purchased at school in the present study. Unfortunately, we were not able to ascertain the source of the foods consumed and thus were unable to examine the extent to which foods consumed at school were brought from home *v*. purchased elsewhere. Future iterations of Mexico’s National Health and Nutrition Survey should take into account where foods are acquired (or purchased) as well as where they are consumed. It is also important to note that in addition to being both a source and a location of food intake for children, schools serve an important role in providing nutrition information to children. In 2010, the Mexican Secretariat of Health and the Secretariat of Public Education issued guidelines on foods and beverages allowed for sale and recommended to be consumed in schools^(^ ^24^ ^)^. These guidelines provide nutritional criteria for breakfast, lunch and snacks consumed at school and encourage, for example, the daily consumption of fruits and vegetables, plain drinking-water and consumption of whole grains^(^ ^24^ ^)^.

After school, the street was the most common location of away-from-home food intake. Previous research has found that, similar to other developing countries, street vendors are especially important sources of dietary intake^(^ ^25^ ^,^ ^26^ ^)^, with a study using Mexico’s 2006 National Health and Nutrition Survey showing that of adults aged >20 years, 32 % consumed meals, 37 % consumed snacks and 54 % consumed drinks from street vendors at least once monthly^(^ ^27^ ^)^. In our study, the prevalence of consuming foods in the street was still somewhat low (13–14 %), but this survey reflects only a single day of intake and it is possible that more children would report intake of street foods if a longer-term dietary assessment was conducted. A second important question relates to what foods and beverages are sold by street vendors and the degree to which these street vendors contribute to foods consumed at or after school, as the food retail environment around schools will influence the nutritional quality of what children buy and eat^(^ ^28^ ^)^. Finally, while away-from-home food intake from restaurants and other locations remained low across age groups, a systematic review of multiple countries showed that the energy contribution from these foods increased into adolescence, suggesting that shifts towards more away-from-home foods may be an issue to monitor in this population as well^(^ ^4^ ^)^.

Our study also found a positive association between SES, total daily energy intake and away-from-home food intake, but no relationship between urbanicity and total or away-from-home food intake. There is currently a dearth of evidence about the association between these sociodemographic factors and diet quality among children, but a previous study using Mexico’s 2006 National Health and Nutrition Survey found that among adults, urban households and those with higher education had higher away-from-home food expenditures^(^ ^27^ ^)^. Studies of the nutrition transition tend to show that as countries develop, higher urbanicity is associated with more varied diets, but that the burden of low diet quality shifts to the urban poor as diets move away from fresh fruits and vegetables, pulses, potatoes and other staples to fast and convenience foods marked by higher sugar, salt and fat content^(^ ^29^ ^,^ ^30^ ^)^. Future work should continue to monitor shifts in eating location and dietary quality by urbanicity and SES, as well as how these factors influence weight gain over time, as studies of Mexican adults have already noted that disparities in obesity vary by both SES and urbanicity^(^ ^31^ ^,^ ^32^ ^)^.

Finally, the sensitivity analysis found that children excluded from main analyses due to missing weight status were more likely to be high SES and, among 2–5-year-olds, have lower total and at-home daily energy intakes compared with those with complete information on weight status. Inclusion of this sample of children with missing weight status in our main analyses may have strengthened the positive association between high SES and energy consumed away from home among 2–5-year-olds, as the excluded sample had a higher percentage of energy from away-from-home foods as well as a higher proportion of high SES. However, because those with missing weight status comprised such a small sample of the population, inclusion of this sample would be unlikely to change our overall findings.

### Limitations

As previously noted, a major limitation was the inability to accurately classify the source from which foods were purchased or obtained. This is important not only for understanding food intake in schools, but also for understanding the overall intake of pre-prepared foods, including the degree to which foods purchased away from home are consumed at home (take-out, delivery). First, understanding the extent to which food stores are a source of purchased foods is important, as SSB and highly energy-dense non-essential foods purchased at stores were subject to taxation in Mexico as of 1 January 2014. In addition, it would be useful to understand whether foods consumed at home were home-prepared or processed, packaged or otherwise pre-prepared, which may be important indictors of nutritional quality, with some processed foods tending to be of poorer nutritional quality^(^ ^33^ ^,^ ^34^ ^)^.

In addition, we were unable to explicitly examine foods consumed at daycare centres. This could be important, especially for younger children, as previous research in Mexico has found that although daycare centre menus met minimum MyPlate recommendations for each food category except for whole grains, for children aged 48–72 months, menus included excessive high-energy beverages including full-fat milk, fruit juice and SSB, and overall excess energy^(^ ^35^ ^)^.

While it would be useful to examine the association between away-from-home food intake and overweight and obesity, especially considering the high prevalence in this population, the cross-sectional nature of the survey precludes causal examination of this relationship. Future longitudinal work will be needed to understand first if away-from-home foods increase over time, and also whether intake location or source is linked to excess energy, poorer dietary quality and weight gain over time. It will also be important to consider selection issues associated with away-from-home eating, as previous work has found that children who eat more away-from-home foods also eat less healthy diets at home^(^ ^36^ ^)^. Finally, in understanding diet–disease associations, a single 24 h recall as used in the present study would likely be inadequate to characterize individual intakes of episodically consumed foods, including those consumed at less-frequented locations such as restaurants or sports arenas.

## Conclusion

Mexican children consumed the majority of daily energy at home, although away-from-home food intake increased with age and with SES. Away-from-home food intake included both healthy and unhealthy foods and did not disproportionately contribute to SoFAS intake. Future work will be needed to monitor potential increases of away-from-home food intake as Mexico continues to develop and to evaluate the nutritional quality of foods consumed across locations, especially in daycare centres and schools.

## Supplementary material

For supplementary material accompanying this paper visit https://doi.org/10.1017/S1368980016002196. click here to view supplementary material
