# Supplementary material for: The contribution of at-home and away-from-home food to dietary intake among 2–13-year-old Mexican children
Source: Public Health Nutr. 2016 Sep 9;20(14):2559–68. doi: 10.1017/S1368980016002196 (PMC5344791; doi:10.1017/S1368980016002196)
Supplement: Supplementary file 1 [file S1368980016002196sup001.docx]

**Online supplementary material**

| Supplemental Table 1. Percent consumers and mean caloric intake by specific eating locations^a^ | | | | | | | | | | | | | | | | |
| --- | --- | --- | --- | --- | --- | --- | --- | --- | --- | --- | --- | --- | --- | --- | --- | --- |
|  | Home | | | School | | | Restaurant | | | Street | | | Other | | | |
|  | % con-sumers | Mean % kcal | SE | % con-sumers | Mean % kcal | SE | % con-sumers | Mean % kcal | SE | % con-sumers | Mean % kcal | SE | % con-sumers |  | Mean % kcal | SE |
| Age |  |  |  |  |  |  |  |  |  |  |  |  |  |  |  |  |
| 2-5 y (ref) | 99 | 89.0 | 0.7 | 22 | 5.9 | 0.5 | 1 | 0.5 | 0.1 | 14 | 3.0 | 0.3 | 5 |  | 1.6 | 0.3 |
| 6-13 y | 99 | 81.9 | 0.7 | 43 | 12.7 | 0.6 | 3 | 1.1 | 0.2 | 13 | 2.8 | 0.3 | 5 |  | 1.5 | 0.2 |
| Sex |  |  |  |  |  |  |  |  |  |  |  |  |  |  |  |  |
| Male (ref) | 99 | 85.4 | 0.8 | 36 | 10.1 | 0.7 | 2 | 0.8 | 0.2 | 11 | 2.4 | 0.3 | 5 |  | 1.4 | 0.2 |
| Female | 99 | 82.7 | 0.7 | 38 | 11.2 | 0.6 | 3 | 1.0 | 0.2 | 15 | 3.4 | 0.4 | 5 |  | 1.6 | 0.3 |
| Region |  |  |  |  |  |  |  |  |  |  |  |  |  |  |  |  |
| South (ref) | 100 | 87.0 | 0.7 | 31 | 9.1 | 0.7 | 2 | 0.8 | 0.2 | 9 | 1.8 | 0.3 | 5 |  | 1.3 | 0.2 |
| Central | 99 | 82.2 | 0.8 | 38 | 11.3 | 0.7 | 3 | 1.1 | 0.2 | 17 | 3.8 | 0.4 | 5 |  | 1.6 | 0.3 |
| North | 99 | 84.2 | 1.0 | 38 | 9.8 | 0.6 | 3 | 0.9 | 0.2 | 12 | 3.3 | 0.8 | 6 |  | 1.8 | 0.4 |
| Mexico City | 99 | 81.0 | 2.2 | 48 | 13.8 | 2.2 | 1 | 0.6 | 0.3 | 15 | 3.0 | 0.8 | 6 |  | 1.6 | 0.5 |
| Urbanicity |  |  |  |  |  |  |  |  |  |  |  |  |  |  |  |  |
| Rural (ref) | 100 | 86.0 | 0.8 | 31 | 9.6 | 0.7 | 1 | 0.4 | 0.1 | 11 | 2.3 | 0.3 | 5 |  | 1.7 | 0.4 |
| Urban | 99 | 83.2 | 0.7 | 39 | 11.1 | 0.6 | 3 | 1.1 | 0.2 | 14 | 3.1 | 0.3 | 5 |  | 1.4 | 0.2 |
| Weight status |  |  |  |  |  |  |  |  |  |  |  |  |  |  |  |  |
| Normal weight (ref) | 99 | 85.1 | 0.6 | 34 | 9.7 | 0.5 | 2 | 0.7 | 0.1 | 13 | 2.9 | 0.3 | 5 |  | 1.5 | 0.2 |
| Overweight/obese | 100 | 80.7 | 1.2 | 45 | 13.5 | 1.2 | 3 | 1.4 | 0.3 | 12 | 2.7 | 0.4 | 6 |  | 1.6 | 0.4 |
| Socio-economic status |  |  |  |  |  |  |  |  |  |  |  |  |  |  |  |  |
| Lowest tertile (ref) | 100 | 88.5 | 0.7 | 27 | 8.5 | 0.7 | 0 | 0.1 | 0.1 | 10 | 1.9 | 0.2 | 3 |  | 0.9 | 0.2 |
| Middle tertile | 99 | 82.6 | 0.9 | 40 | 11.2 | 0.7 | 2 | 0.6 | 0.1 | 15 | 3.9 | 0.5 | 6 |  | 1.7 | 0.3 |
| Highest tertile | 99 | 80.9 | 1.1 | 45 | 12.4 | 1.0 | 5 | 2.1 | 0.4 | 13 | 2.8 | 0.4 | 7 |  | 1.9 | 0.3 |
| Eating occasion, per capita |  |  |  |  |  |  |  |  |  |  |  |  |  |  |  |  |
| Breakfast | 74 | 19.4 | 0.4 | 8 | 2.2 | 0.2 | 0 | 0.1 | 0.1 | 1 | 0.3 | 0.1 | 1 |  | 0.3 | 0.1 |
| Almuerzo | 26 | 7.3 | 0.3 | 17 | 4.6 | 0.3 | 0 | 0.1 | 0.0 | 1 | 0.2 | 0.1 | 1 |  | 0.2 | 0.1 |
| Lunch | 83 | 26.6 | 0.5 | 4 | 1.1 | 0.1 | 1 | 0.4 | 0.1 | 2 | 0.5 | 0.1 | 2 |  | 0.5 | 0.1 |
| Dinner | 79 | 19.1 | 0.4 | 1 | 0.1 | 0.0 | 1 | 0.2 | 0.1 | 2 | 0.4 | 0.1 | 1 |  | 0.1 | 0.0 |
| Total snacks | 57 | 11.6 | 0.4 | 14 | 2.6 | 0.3 | 0 | 0.1 | 0.0 | 10 | 1.4 | 0.1 | 3 |  | 0.4 | 0.1 |
| Eating occasion, per consumer^b^ | |  |  |  |  |  |  |  |  |  |  |  |  |  |  |  |
| Breakfast | 88 | 23.1 | 0.4 | 9 | 2.7 | 0.3 | 0 | 0.2 | 0.1 | 1 | 0.4 | 0.1 | 2 |  | 0.4 | 0.1 |
| Almuerzo | 58 | 16.1 | 0.6 | 39 | 10.1 | 0.5 | 1 | 0.2 | 0.1 | 2 | 0.4 | 0.1 | 1 |  | 0.4 | 0.1 |
| Lunch | 91 | 29.1 | 0.5 | 4 | 1.2 | 0.2 | 1 | 0.4 | 0.1 | 2 | 0.6 | 0.1 | 2 |  | 0.6 | 0.1 |
| Dinner | 96 | 23.2 | 0.4 | 1 | 0.2 | 0.0 | 1 | 0.2 | 0.1 | 2 | 0.5 | 0.1 | 1 |  | 0.1 | 0.1 |
| Total snacks | 82 | 16.6 | 0.4 | 20 | 3.7 | 0.4 | 0 | 0.1 | 0.0 | 14 | 2.0 | 0.2 | 4 |  | 0.5 | 0.1 |
| ^a^ Sample sizes for demographics are based on all respondents with non-missing values on age, sex, urbanicity, and SES. Sample sizes for eating occasion are those who consumed >0 kcal during the eating occasion. ^b^ Percent consumer defined as the percent of children who ate food at or from a given source or location at that eating occasion child, of those consumed >0 kcal at the eating occasion | | | | | | | | | | | | | | | | |

| Supplemental Table 2. Descriptive statistics for excluded children (2-13y with missing weight status) compared to included children (2-13y without missing weight status) | | | | | | | | | | | | | | | | | |
| --- | --- | --- | --- | --- | --- | --- | --- | --- | --- | --- | --- | --- | --- | --- | --- | --- | --- |
|  |  |  |  |  |  |  |  |  |  |  |  |  |  |  |  |  |  |
| Weight Status missing (n=254) |  |  |  | | |  | At home | | | | |  | Away-from-home | | | | |
|  |  |  | Mean (SE) per capita total daily calories | | |  | % consumers |  | Mean per capita daily calories | | |  | % consumers |  | Mean per capita daily calories | | |
|  | % of sample |  | Mean |  | SE |  |  |  | Mean |  | SE |  |  |  | Mean |  | SE |
| Age |  |  |  |  |  |  |  |  |  |  |  |  |  |  |  |  |  |
| 2-5 y (ref) | 52 |  | **1345** |  | 57 |  | 99 |  | **1157** |  | 63 |  | 34 |  | 188 |  | 45 |
| 6-13 y | 48 |  | 2022 |  | 136 |  | 99 |  | 1628 |  | 139 |  | 67 |  | 394 |  | 51 |
| Sex |  |  |  |  |  |  |  |  |  |  |  |  |  |  |  |  |  |
| Male (ref) | 58 |  | 1825 |  | 146 |  | **98** |  | 1484 |  | 145 |  | 56 |  | 340 |  | 47 |
| Female | 42 |  | 1700 |  | 107 |  | 100 |  | 1412 |  | 109 |  | 52 |  | 288 |  | 56 |
| Region |  |  |  |  |  |  |  |  |  |  |  |  |  |  |  |  |  |
| South (ref) | **23** |  | 1801 |  | 168 |  | **97** |  | 1551 |  | 175 |  | 42 |  | 250 |  | 66 |
| Central | 44 |  | 1621 |  | 93 |  | 100 |  | 1329 |  | 91 |  | 49 |  | 292 |  | 65 |
| North | 28 |  | 1771 |  | 123 |  | 100 |  | 1476 |  | 127 |  | 54 |  | 295 |  | 58 |
| Mexico City | 6 |  | 2076 |  | 428 |  | 97 |  | 1564 |  | 452 |  | 85 |  | 512 |  | 100 |
| Urbanicity |  |  |  |  |  |  |  |  |  |  |  |  |  |  |  |  |  |
| Rural (ref) | 32 |  | 1745 |  | 170 |  | **97** |  | 1480 |  | 194 |  | 35 |  | 266 |  | 83 |
| Urban | 68 |  | 1777 |  | 113 |  | 99 |  | 1443 |  | 110 |  | 60 |  | 334 |  | 40 |
| Socio-economic status |  |  |  |  |  |  |  |  |  |  |  |  |  |  |  |  |  |
| Lowest tertile (ref) | 33 |  | 1870 |  | 224 |  | **98** |  | 1550 |  | 230 |  | **54** |  | 320 |  | 65 |
| Middle tertile | **30** |  | 1668 |  | 94 |  | 100 |  | 1419 |  | 74 |  | 46 |  | 249 |  | 50 |
| Highest tertile | **37** |  | 1737 |  | 98 |  | 99 |  | 1385 |  | 98 |  | 58 |  | 352 |  | 60 |
|  |  | | | | | | | | | | | | | | | | |
| Weight Status non-missing (n=4773) |  |  |  | | |  | At home | | | | |  | Away-from-home | | | | |
|  |  |  | Mean (SE) per capita total daily calories | | |  | %  consumers |  | Mean per capita daily calories | | |  | %  consumers |  | Mean per capita daily calories | | |
|  | % of sample |  | Mean |  | SE |  |  |  | Mean |  | SE |  |  |  | Mean |  | SE |
| Age |  |  |  |  |  |  |  |  |  |  |  |  |  |  |  |  |  |
| 2-5 y (ref) | 40 |  | 1494 |  | 23 |  | 99 |  | 1333 |  | 24 |  | 37 |  | 161 |  | 10 |
| 6-13 y | 60 |  | 1907 |  | 24 |  | 99 |  | 1549 |  | 23 |  | 56 |  | 359 |  | 15 |
| Sex |  |  |  |  |  |  |  |  |  |  |  |  |  |  |  |  |  |
| Male (ref) | 51 |  | 1854 |  | 28 |  | 99 |  | 1565 |  | 28 |  | 48 |  | 289 |  | 18 |
| Female | 49 |  | 1712 |  | 23 |  | 99 |  | 1403 |  | 23 |  | 52 |  | 310 |  | 14 |
| Region |  |  |  |  |  |  |  |  |  |  |  |  |  |  |  |  |  |
| South (ref) | 36 |  | 1756 |  | 28 |  | 100 |  | 1506 |  | 26 |  | 41 |  | 251 |  | 16 |
| Central | 36 |  | 1726 |  | 28 |  | 99 |  | 1399 |  | 28 |  | 53 |  | 327 |  | 18 |
| North | 23 |  | 1867 |  | 40 |  | 99 |  | 1571 |  | 39 |  | 52 |  | 295 |  | 19 |
| Mexico City | 5 |  | 1853 |  | 74 |  | 99 |  | 1499 |  | 69 |  | 60 |  | 355 |  | 49 |
| Urbanicity |  |  |  |  |  |  |  |  |  |  |  |  |  |  |  |  |  |
| Rural (ref) | 39 |  | 1759 |  | 29 |  | 100 |  | 1489 |  | 29 |  | 42 |  | 270 |  | 17 |
| Urban | 61 |  | 1793 |  | 24 |  | 99 |  | 1481 |  | 23 |  | 53 |  | 312 |  | 15 |
| Socio-economic status |  |  |  |  |  |  |  |  |  |  |  |  |  |  |  |  |  |
| Lowest tertile (ref) | 38 |  | 1717 |  | 30 |  | 100 |  | 1505 |  | 30 |  | 37 |  | 212 |  | 15 |
| Middle tertile | 36 |  | 1807 |  | 32 |  | 99 |  | 1486 |  | 34 |  | 53 |  | 320 |  | 16 |
| Highest tertile | 27 |  | 1826 |  | 36 |  | 99 |  | 1457 |  | 32 |  | 60 |  | 369 |  | 27 |
| **Bold** indicates the mean for the weight status missing sample is different from the weight status non-missing sample using 2-sample t-test at p<0.05, or percent is different using chi-square at p<0.05. | | | | | | | | | | | | | | | | | |
